# Neural Synaptic Simulation Based on ZnAlSnO Thin-Film Transistors

**DOI:** 10.3390/mi16091025

**Published:** 2025-09-07

**Authors:** Yang Zhao, Chao Wang, Laizhe Ku, Liang Guo, Xuefeng Chu, Fan Yang, Jieyang Wang, Chunlei Zhao, Yaodan Chi, Xiaotian Yang

**Affiliations:** 1 Key Laboratory of Architectural Cold Climate Energy Management, Ministry of Education, Jilin Jianzhu University, Changchun 130118, China; 15567080162@163.com (Y.Z.); klz0807@163.com (L.K.); guoliang@jlju.edu.cn (L.G.); stone2009@126.com (X.C.); ctpnxn@163.com (F.Y.); jieyang202309@163.com (J.W.); zhaochunlei@jlju.edu.cn (C.Z.); chiyaodan@jlju.edu.cn (Y.C.); 2 School of Electrical and Computer Science, Jilin Jianzhu University, Changchun 130118, China; 3 Department of Basic Science, Jilin Jianzhu University, Changchun 130118, China; 4 Department of Chemistry, Jilin Normal University, Siping 136000, China

**Keywords:** neuromorphic computation, neural synaptic device, aluminum zinc tin oxide (znalsno), thin-film transistor

## Abstract

In the era of artificial intelligence, neuromorphic devices that simulate brain functions have received increasingly widespread attention. In this paper, an artificial neural synapse device based on ZnAlSnO thin-film transistors was fabricated, and its electrical properties were tested: the current-switching ratio was 1.18 × 10^7^, the subthreshold oscillation was 1.48 V/decade, the mobility was 2.51 cm^2^V^−1^s^−1^, and the threshold voltage was −9.40 V. Stimulating artificial synaptic devices with optical signals has the advantages of fast response speed and good anti-interference ability. The basic biological synaptic characteristics of the devices were tested under 365 nm light stimulation, including excitatory postsynaptic current (EPSC), paired-pulse facilitation (PPF), short-term plasticity (STP), and long-term plasticity (LTP). This device shows good synaptic plasticity. In addition, by changing the gate voltage, the excitatory postsynaptic current of the device at different gate voltages was tested, two different logical operations of “AND” and “OR” were achieved, and the influence of different synaptic states on memory was simulated. This work verifies the application potential of the device in the integrated memory and computing architecture, which is of great significance for promoting the high-quality development of neuromorphic computing hardware.

## 1. Introduction

Since Turing proposed the concept of the Turing machine computing model in 1936 [1], computers have experienced rapid development. However, with the development of the times, the existing computers, due to their disadvantages such as low transmission efficiency and slow transmission speed, have increasingly failed to meet the requirements of the times. The human brain has a large number of neurons and synapses, enabling it to process information in parallel and operate at high speed [2,3,4]. Therefore, neuromorphic computing inspired by the human brain has developed rapidly. And synapses are important structures of the human brain nervous system. So, artificial neural synaptic devices are of great significance for promoting neuromorphic computing.

In recent years, the research on neuromorphic devices based on metal oxide thin-film transistors has gradually increased [5,6,7,8]. In 2021, Cho SI et al. fabricated InZnO (IZO)/InGaZnO (IGZO) synaptic transistors and simulated various synaptic characteristics, including excitatory postsynaptic current, paired pulse promotion, enhancement, and inhibition [9]. In 2021, He Yongli from Nanjing University fabricated IGZO and InSnO (ITO) oxide thin-film transistors and successfully simulated functions such as double-pulse facilitation of biological synapses [10]. In the same year, Liu Rui from Nanjing University fabricated the IZO oxide thin-film transistor and successfully simulated the process of the transition from short-range memory to long-range memory. This process could be simulated by applying electrical pulses to the transistor gate and using channel currents [11]. In 2022, Jang Y et al. studied synaptic transistors based on amorphous IGZO and described the fundamental principles of neuromorphic computing, neuronal circuits, and synaptic devices [12]. In 2022, Zhang Guifa from Jiangsu University fabricated IGZO oxide thin-film transistors and successfully simulated the excitable postsynaptic current characteristics of biological synapses using electrical pulses [13].

InGaZnO [14,15,16] and ZnAlSnO (AZTO) [17,18] are both typical oxide semiconductors. However, compared with indium and gallium, aluminum and tin are not rare elements [19]. They can both be produced in large quantities and are inexpensive. Moreover, neither of these two elements is toxic and they are environmentally friendly. The research on artificial synaptic devices for neuromorphic simulation mainly uses electrical signals [20,21,22]. The research on neuromorphic devices of AZTO thin-film transistors is relatively scarce. Moreover, optical signals have advantages such as strong anti-interference ability and fast transmission speed compared with electrical signals [23,24,25,26]. Based on this, in this paper, AZTO thin-film transistor type artificial synaptic devices were fabricated. The electrical performance of the devices was tested and the basic characteristics of biological synapses, such as excitatory postsynaptic current (EPSC), short-term plasticity (STP), paired-pulse facilitation (PPF), and long-term plasticity (LTP), were simulated using light pulses. By adjusting the gate voltage, the excitatory postsynaptic current of the device at different gate voltages was tested, different logical operations were achieved, and the memory levels of the brain under different states were simulated.

## 2. Experiment

### 2.1. Device Fabrication

First, the substrate with a self-contained 285 nm thick SiO_2_ insulating layer was ultrasonically cleaned for 10 min each in acetone, alcohol, and deionized water to remove impurities on the substrate surface. Then, it was blown dry with nitrogen, homogenized using a coater, and dried on a dryer for 5 min. A lithography was performed using a mask and American ABM Company Ultraviolet lithography machine, and it was dried again on a dryer for 5 min. Place the sample on the photolithography machine and expose it naked for 3 s. After completion, put the sample into sodium hydroxide solution and then pure water. Repeat this step for development. After drying, observe it under a microscope. Secondly, AZTO films were deposited byPVD75 Magnetron Sputtering System by Kurt J. Lesker, USA. Then, they were washed with acetone, alcohol, and deionized water for 3 min each, and dried with nitrogen. The samples were placed in German-made RTP-100 rapid thermal processor for annealing. After annealing was completed, secondary photolithography was carried out using a mask and a photolithography machine. After photolithography was completed, the samples were placed in sodium hydroxide solution and then pure water for secondary development. After the development was completed, it was dried with nitrogen gas. Finally, the sample was placed in LN-2063SA organic vapor deposition system manufactured by Shenyang Lining Vacuum Technology Research Institute, Chinato prepare the Al electrode. At this point, the AZTO artificial synaptic device has been successfully fabricated.

### 2.2. Device Structure and Characterization

**Figure 1:**
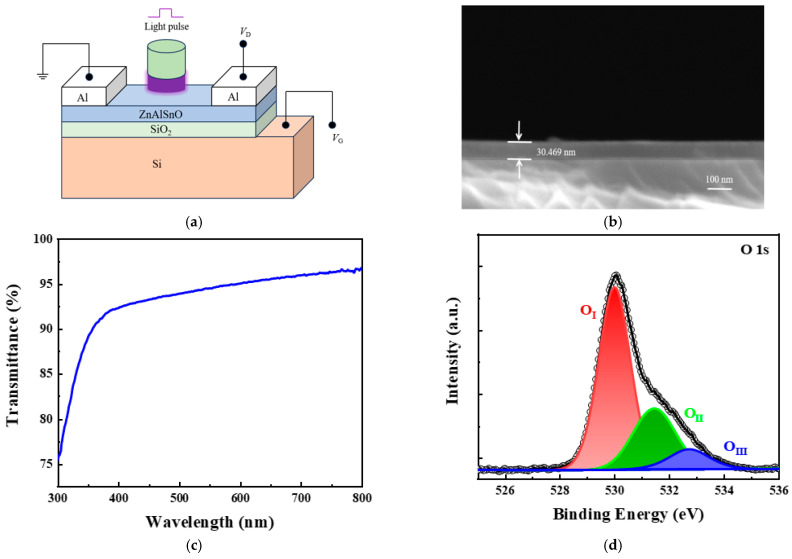

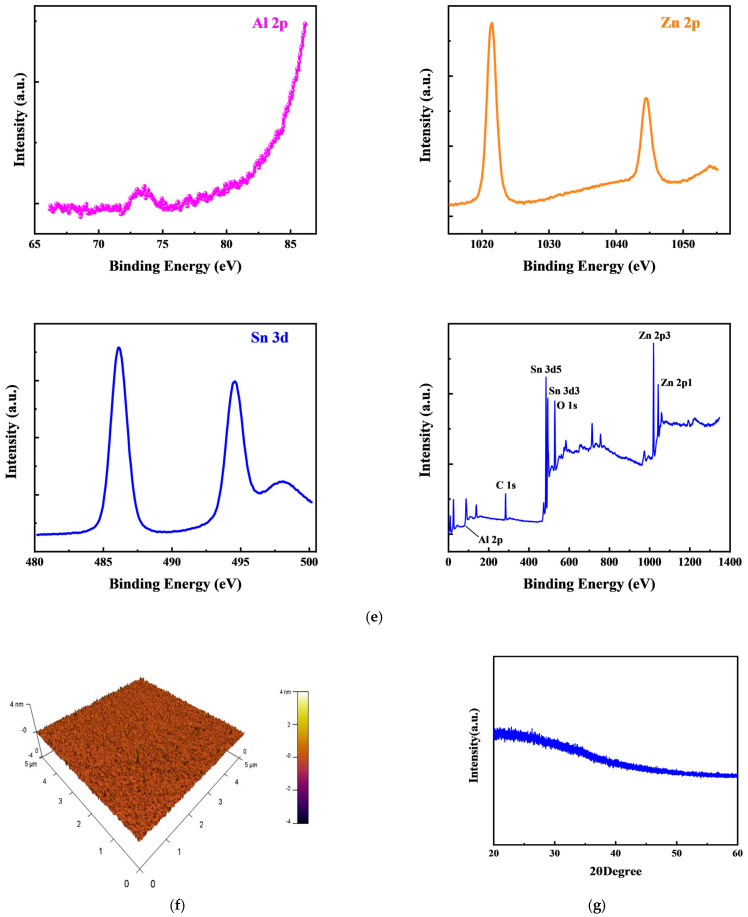
Device structure and characterization. (**a**) Schematic diagram of the artificial synaptic device structure. (**b**) SEM image of the cross-section of the AZTO film. (**c**) Transmission spectrum of the AZTO film. (**d**) XPS spectrum of O-1s. (**e**) XPS spectra of Al 2p, Zn 2p, Sn 3d, and full XPS spectra. (**f**) AFM image of AZTO. (**g**) XRD analysis of AZTO.

The structure of the AZTO artificial synaptic device is shown in Figure 1a. The thickness of the AZTO film was measured to be approximately 30 nm using a scanning electron microscope, as shown in Figure 1b. The transmittance of the film was tested using a Shimadzu UV-2600 UV-Vis spectrophotometer, as shown in Figure 1c. Figure 1d shows the XPS spectrum of O-1s. The spectrum of O-1s is divided into three peaks, located at 529.99 eV (O_I_), 531.44 eV (O_II_), and 532.76 eV (O_III_), representing lattice oxygen, oxygen vacancies, and adsorbed oxygen on the surface, respectively. Their respective contents are 61.77%, 30.29%, and 7.94%. Figure 1e displays the XPS spectra of Al 2p, Zn 2p, Sn 3d, and full XPS spectra. The contents of C 1s, Al 2p, Zn 2p, Sn 3d, and O 1s are 31.75%, 0.75%, 20.21%, 11.92%, and 35.38%, respectively. Figure 1d and e were tested using the ESCALAB 250Xi equipment manufactured by Thermo Fisher Scientific. Figure 1f presents the AFM image of AZTO. It was tested using an MFP-3D atomic force microscope manufactured by Oxford Instruments in the UK. The surface area of the film selected for testing was 5 μm × 5 μm. The surface morphology of the film was tested using atomic force microscopy (AFM), and the film surface was found to be uniform overall. The roughness measured by AFM was 161.46 pm, indicating that the film surface was relatively flat. Figure 1g shows the XRD test results for the sample, revealing no diffraction peaks in the fabricated device. This indicates that the AZTO film exhibits an amorphous structure and that AZTO exists in an amorphous state. Figure 1g was tested using a D8 DISCOVER X-ray diffractometer manufactured by Bruker Corporation of Germany.

### 2.3. The Electrical Properties of AZTO-TFT

**Figure 2:**
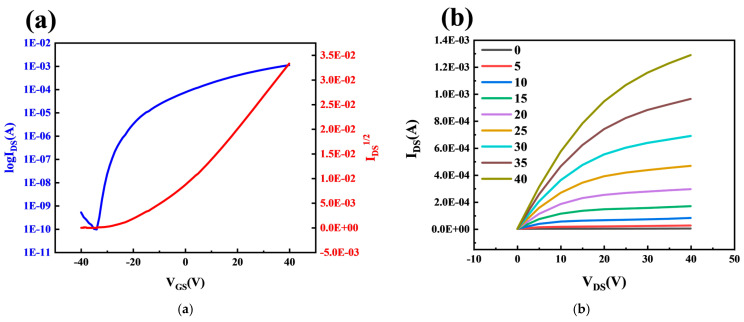
(**a**) Transfer characteristic curve of AZTO-TFT. (**b**) Output characteristic curve of AZTO-TFT.

The electrical properties of AZTO-TFT were analyzed and tested using a semiconductor parameter meter. Figure 2a shows the transfer characteristic curve of AZTO-TFT. The transfer characteristic curve is a graph showing the relationship between the channel current and the gate voltage. When testing the transfer characteristic curve, the variation range of the gate voltage was −40–40 V. The drain voltage is 20 V. Figure 2b shows the output characteristic curve of AZTO-TFT. The output characteristic curve is the relationship graph of the channel current varying with the drain voltage. When testing the output characteristic curve, the conditions of the channel current varying with the drain voltage under the gate voltage in nine cases were tested, respectively. The variation range of the gate voltage was 0–40 V, with a step size of 5 V. The variation range of the drain voltage was 0–40 V.

The principle behind the transfer characteristic curve of a TFT is that a conductive channel is induced in the active layer by the gate voltage. When *V*_GS_ exceeds the threshold voltage, a conductive channel forms in the active layer, and *I*_DS_ increases with increasing *V*_GS_. As *V*_GS_ continues to increase, *I*_DS_ approaches saturation. As shown in Figure 2a, as the gate voltage increases, *I*_DS_ increases rapidly, and the device exhibits excellent switching characteristics. It can be seen from Figure 2b that when the gate voltage is higher than the threshold voltage, the channel current first shows a linear increasing trend with the increase of *V*_DS_. As V_D_ continues to increase, the channel current tends to saturation, meaning that the channel current no longer increases with the increase of V_DS_.

Combined with the characteristic curve of the thin-film transistor, the off-state current, on-state current, switching ratio, sub-threshold swing, carrier mobility, and threshold voltage of the device can be calculated according to Equations (1)–(3).

(1) $$\mu_{\mathrm{SAT}}=\frac{2L}{WC_{i}}\;{\left(\frac{\partial \sqrt{I_{\mathrm{DS}}}}{\partial V_{\mathrm{GS}}}\right)}^{2}$$

(2) $$I_{\mathrm{ON}}/I_{\mathrm{OFF}}\;=\;\frac{{\left(I_{\mathrm{DS}}\right)}_{\max}}{{\left(I_{\mathrm{DS}}\right)}_{\min}}$$

(3) $$\mathrm{SS}\;=\;\frac{dV_{\mathrm{GS}}}{d(\log I_{\mathrm{DS}})}$$

In the equation, *C_i_* represents the area capacitance of the gate insulation layer, *W* is the channel width of the active layer, *L* is the channel length of the active layer, *V*_GS_ indicates the voltage applied to the gate, and *I*_DS_ is the channel current.

It can be known through calculation that the current-switching ratio of the device is 1.18 × 10^7^, the subthreshold swing is 1.48 V/decade, the mobility is 2.51 cm^2^V^−1^s^−1^, and the threshold voltage is −9.40 V.

### 2.4. Synaptic Characteristic Simulation

The human brain is a highly complex network composed of a large number of neurons [27,28,29]. Numerous neurons are connected to other neurons through synapses, and neuron cells are composed of dendrites, cell bodies, and axons. The axon terminals of neurons have many tiny branches. The swollen part at the end of each branch is the synaptic vesicle. The synaptic vesicle of the previous neuron is connected to the cell body or dendrite of the next neuron to form a synapse. The connection strength between neurons is called synaptic weight. Synaptic plasticity refers to the property that the connection strength between neurons can be regulated. Synapses consist of three parts: the presynaptic membrane, the synaptic cleft, and the postsynaptic membrane. The axon terminals contain many synaptic vesicles. When the brain is stimulated by signals, the synaptic vesicles are stimulated and fuse with the presynaptic membrane, releasing neurotransmitters (converting electrical signals into chemical signals). Neurotransmitters then bind to receptors on the postsynaptic membrane through the synaptic cleft (converting chemical signals into electrical signals). It triggers potential changes in the postsynaptic membrane. After the signal transmission is completed, the neurotransmitter separates from the receptor.

**Figure 3:**
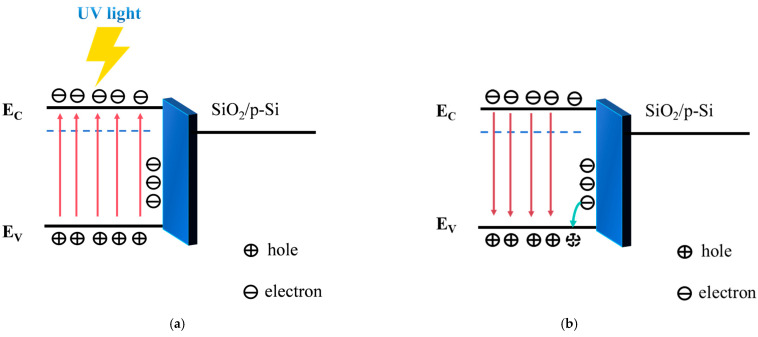
(**a**) Schematic diagram of carrier behavior under ultraviolet irradiation. (**b**) Schematic diagram of carrier behavior after ultraviolet irradiation.

The bandgap refers to the energy difference between the lowest point of the conduction band and the highest point of the valence band. The energy of incident light must exceed the semiconductor’s bandgap to excite electron transitions. Ultraviolet light has shorter wavelengths and higher energy than visible light. Materials with larger bandgaps typically absorb ultraviolet light because its high energy is sufficient to enable electrons to jump across the bandgap into higher energy levels. When ultraviolet light is incident on a device, electrons in the valence band absorb the energy of incident photons and transition to the conduction band. The fundamental principle and mechanism underlying synapse characteristic simulation is essentially the persistent photoconductive effect in semiconductor materials. As a wide-bandgap semiconductor material, AZTO possesses the ability to absorb ultraviolet light, excite electron transitions, and generate a persistent photoconductive effect, making it suitable as the active layer for synapse devices. Figure 3 illustrates the band structure diagram under and after ultraviolet irradiation. After electron transitions occur, vacancies are left in the valence band. These vacancies are photogenerated holes, and photogenerated carriers are electron–hole pairs composed of photogenerated electrons and photogenerated holes. XPS tests have shown the existence of oxygen vacancies in AZTO. During the illumination process, Oxygen vacancies will capture photogenerated holes [26,30,31]. Due to the capture effect of interface charges, some photogenerated electrons will accumulate at the interface, and another part will be replenished into the transistor’s conductive channel, thus increasing the conductivity. Due to the continuous photoconductive effect of semiconductors, even after the light pulse ends, the channel conductivity of the device will not change immediately. The above process can be explained according to Equations (4) and (5):

(4) $$\mathrm{AZTO}\;+\;\mathrm{hv}\;\to \;\mathrm{AZTO}\;+\;h^{+\;}+{\;e}^{-}$$

(5) $$O_{2}\;+\;2V_{O}^{2-}\;+\;4h^{+}\;↔\;2O$$

**Figure 4:**
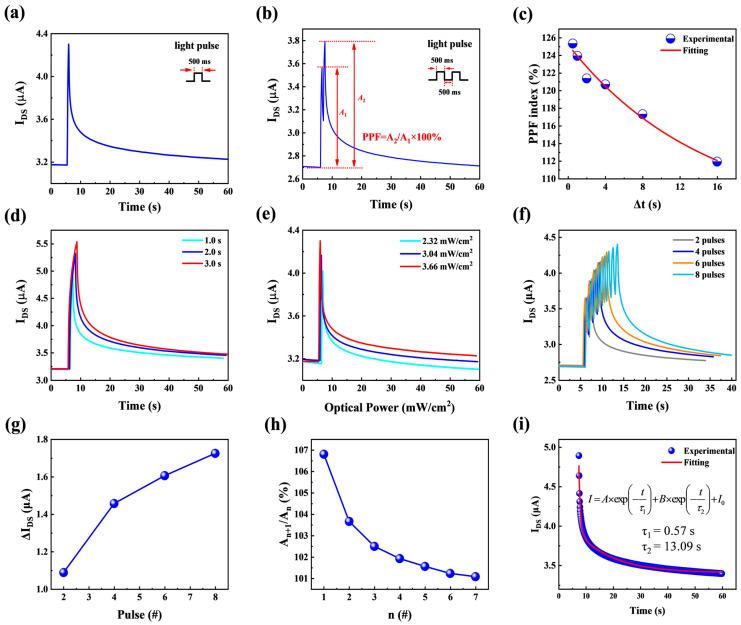
(**a**) Single-pulse light-triggered EPSC of 3.66 mW/cm^2^, with a pulse width of 0.5 s. (**b**) Double-pulse light-triggered PPF, with a pulse width of 0.5 s. (**c**) The relationship between the PPF index and the pulse interval time conforms to the exponential function. (**d**) I_DS_ triggered by single-pulse light with different durations (from 1.0 s to 3.0 s) at an optical power density of 3.66 mW/cm^2^. (**e**) I_DS_ triggered by single-pulse light with different optical power densities. The duration of each optical pulse is 0.5 s. (**f**) For I_DS_ triggered by different numbers of optical pulses, the pulse width is 0.5 s and the pulse interval is 0.5 s. (**g**) Relationship between current change and number of light pulses. (**h**) When the pulse interval is 0.5 s, the ratio of the current peak triggered by the latter pulse to that triggered by the preceding pulse. (**i**) The current attenuation curve triggered by a single optical pulse of 1 s.

For three-terminal devices, the source/drain electrode corresponds to the presynaptic/postsynaptic membrane, the light pulse can be regarded as the action potential acting on the presynaptic, and the photogenerated electrons are equivalent to neurotransmitters. Then, the transistor channel current I_DS_ can be regarded as the excitatory postsynaptic current. The synaptic characteristics of thin-film transistors were tested using a semiconductor parameter meter. The gate voltage was set to 0 V and the source-drain voltage was set to 0.5 V. Use a 365 nm UV LED curing point light source to illuminate the device at an angle, focusing the point light source on the device channel. The distance between the light source and the sample is 2 cm. Then, use a UV radiometer connected to a UVALED-X3 probe to measure the light power density. After applying a light pulse with a duration of 0.5 s and an optical power density of 3.66 mW/cm^2^, the channel current I_DS_ increased from 3.17 μA to 4.30 μA and gradually decreased after the end of the illumination, as shown in Figure 4a. At the beginning, when there was no light pulse stimulation, the current was in the initial state and could be regarded as the resting state of the neuron. During the illumination process, the illumination leads to an increase in the carrier concentration, and the current rapidly increases to the peak, which can be regarded as the action state of the neuron. After the illumination ends, due to the continuous photoconductive effect, the current gradually decreases to the initial value, which can be regarded as the process of recovering from the action state to the resting state. The entire process is similar to EPSC in the short-term plasticity of the synapse. PPF refers to the phenomenon that if the presynaptic membrane is stimulated twice within a short time interval, the response of the postsynaptic membrane induced by the second stimulation is greater than that induced by the first stimulation. As can be seen from Figure 4b, when the device is irradiated with two light pulses with an interval of 0.5 s, the current triggered by the second light pulse is greater than that triggered by the first light pulse. The reason is that after the first light pulse irradiates the device, photogenerated carriers are produced. Due to the continuous photoconductivity effect, it takes a certain amount of time for electrons and holes to return to their initial states. When the time interval between two optical pulses is less than the time required for the carriers to return to their initial state, the carrier concentration when the second optical pulse irradiates the device will be greater than that during the first optical pulse, thereby causing the second current response to be greater than the first current response. The PPF index can be calculated by Equation (6):

(6) $$\mathrm{PPF}\;\mathrm{index}\;=\;\frac{A_{2}}{A_{1}}\;\times \;100\%$$

If the time interval between two optical pulses increases, more carriers will return to their initial state. That is to say, the difference in the amount of current change caused by the two optical pulses will decrease as the time interval increases. The experiment tested six groups of two-pulse generalization indices with time intervals of 0.5 s, 1 s, 2 s, 4 s, 8 s, and 16 s, respectively, and fitted them according to Equation (7):

(7) $$y\;=\;y_{0}\;+\;A\;\times \;\exp(R_{0}\;\times \;x)$$

The relationship between the PPF index and the time interval is shown in Figure 4c. When the interval time is 0.5 s, the PPF index is 125.34%. With the increase in the pulse interval time, the PPF index shows a downward trend. As can be seen from Figure 4c, the six groups of data have a high degree of fit with the fitted curve, which indicates that the PPF index shows an exponential downward trend with the increase in the pulse interval time. The entire process is similar to PPF in the short-term plasticity of synapses. Figure 4d shows the relationship between the channel current and the duration of the light pulse. It can be seen from the figure that as the duration of light exposure increases, the peak value of the current becomes larger and larger, and the final value of the current decline after the same period of time following the peak value of the current is different. It can be known from the figure that the final value of the current increases with the increase in the duration of the light pulse. This is because when a longer duration of light pulse irradiates the device, the electrons in the valence band absorb more energy from the incident photons, generate more carriers, have a higher carrier concentration, and thus have a greater channel current. After the same descent time, the final value of the current is also greater. In other words, the longer the duration of the light pulse, the longer it takes for the current to drop to its initial value. In biology, synaptic weight refers to the connection strength between synapses. Synaptic plasticity is classified into short-term plasticity and long-term plasticity based on the duration of changes in synaptic connection strength. Excitatory postsynaptic current, as an important indicator of synaptic weight change [32], can visually represent the change in synaptic weight. Therefore, the change in current with the light exposure time can indicate that synaptic plasticity also changes with the increase in light exposure time. The light pulse time is regarded as the learning time, and the decrease process of current after the disappearance of the light pulse is regarded as the forgetting process of memory. The magnitude of the current value reflects the level of memory. It can be understood more vividly that the synaptic weight has shown a tendency to transform from short-term plasticity to long-term plasticity. As the light exposure time increases from 1 s to 3 s, the transformation trend becomes more and more obvious. Figure 4e shows the relationship between channel current and optical pulse power density. The principle is similar to that in Figure 4d. It can be seen that as the optical power density increases, the current also increases, and after the same decline time, the final value of the current becomes larger. Figure 4f shows the relationship between the channel current and the number of light pulses. The principle is similar to that in Figure 4b. If the time interval between the previous light pulse and the next light pulse is less than the time required for the carriers to return to their initial state, it will result in the carrier concentration being greater when the device is illuminated by the next light pulse than when it is illuminated by the previous light pulse. This leads to the current increasing with the increase in the number of optical pulses. Figure 4e,f are also simulations of the transformation process from short-term plasticity to long-term plasticity. It can be known from Figure 4d–f that short-term plasticity can be transformed into long-term plasticity through methods such as reinforcement stimulation and continuous training. Figure 4g shows the relationship between the change in current and the number of light pulses. The figure indicates that the change in current increases as the number of light pulses increases. As shown in Figure 4h, the ratio of the current peaks triggered by adjacent pulses decreases gradually as the number of pulses increases. This is because the number of photoelectrons is limited, and the number of photoelectrons generated by subsequent light pulses decreases. This leads to a gradual decrease in the ratio of the current peaks triggered by adjacent pulses in Figure 4h. Figure 4i shows the attenuation curve of the current when the optical pulse time is 1 s, which is fitted using Equation (8):

(8) $$I\;=\;A\;\times \;\exp(-\frac{t}{\tau_{1}})\;+\;B\;\times \;\exp(-\frac{t}{\tau_{2}})\;+\;I_{0}$$

After fitting with a double exponential decay function, the red line in the figure represents the fitted curve, where the parameters τ_1_ is 0.57 s and τ_2_ is 13.09 s. The time constants τ_1_ and τ_2_ correspond, respectively, to intrinsic shallow-level defects and deep-level defects within the active layer material of the device (τ_1_ corresponds to shallow-level defects, where carriers are readily captured, released, and participate in recombination processes; τ_2_ corresponds to deep-level defects, where captured carriers are difficult to release and function as recombination centers). The double-exponential decay of current is intrinsically linked to the material’s inherent defects, which govern carrier recombination pathways. Consequently, variations in τ_1_ and τ_2_ are closely tied to the material’s intrinsic properties rather than dependent on optical power, pulse width, or pulse count.

### 2.5. Simulation of EPSC and Logical Functions Under Different Gate Voltages

**Figure 5:**
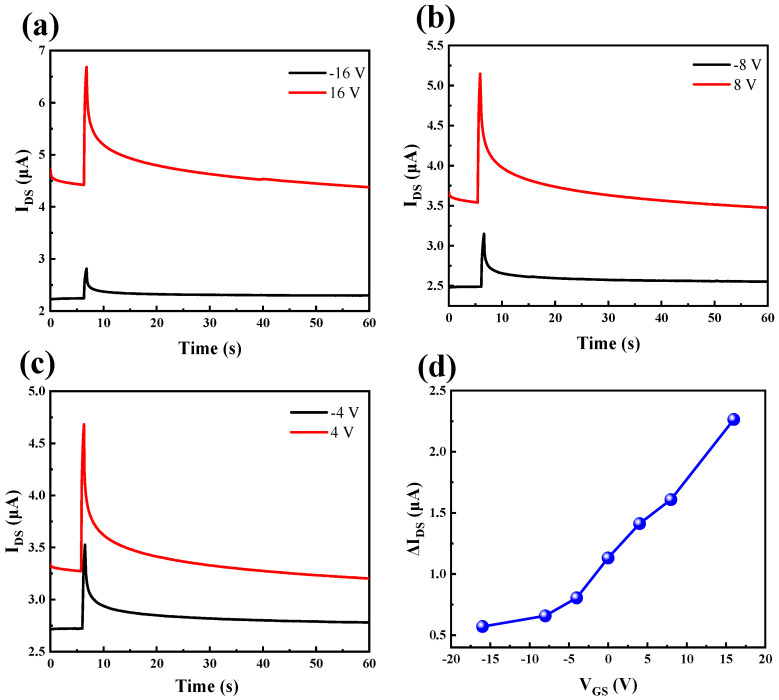
Light pulse-evoked EPSC at different gate voltages: (**a**) −16 V, 16V. (**b**) −8 V, 8V. (**c**) −4 V, 4V. (**d**) Relationship between current change and gate voltage.

To test the influence of different gate voltages on EPSC, the gate voltage was changed, and the device was irradiated with a single optical pulse of an optical power density of 3.66 mW/cm^2^ and a duration of 0.5 s. The EPSC under different gate voltages are shown in Figure 5a–c. Figure 5d shows the relationship between the variation in current and different gate voltages. When the gate voltage increases from −16 V to 16 V, ΔI_DS_ increases from 0.57 μA to 2.26 μA. It can be seen from the figure that the change in current increases with the increase in the gate voltage.

**Figure 6:**
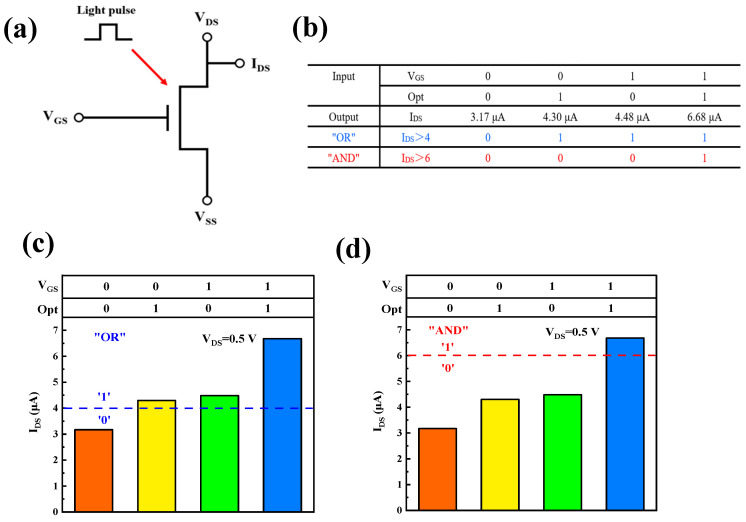
(**a**) Equivalent circuit diagram for “AND” and “OR” logic. (**b**) Truth tables for AND and OR gates, V_DS_: 0.5 V. (**c**) Diagram illustrating the “OR” logical operation. (**d**) Diagram illustrating the “AND” logical operation.

The gate voltage and the light pulse are regarded as two input signals, and the channel current value is regarded as the output signal. Based on the two sets of data of V_GS_ being 0 V and 16 V under the condition that the duration of the optical pulse is 0.5 s in the test, when V_GS_ is 0 V, it is defined as “0”; when V_GS_ is 16 V, it is defined as “1”; a pulse without light is defined as “0”; and a pulse with light is defined as “1”. The output of the device is jointly controlled by two input signals. When there is no input signal, the average value of the dark current is taken as the output. When the gate voltage is zero, the peak current caused by the optical pulse is taken as the output. When the gate voltage is 16 V and there is no light pulse input, the average value of the dark current is taken as the output. When the gate voltage is 16 V and there is an input of optical pulses, the peak value caused by the optical pulses is taken as the output. We can set 4 μA and 6 μA as current thresholds. As shown in Figure 6b, this is the truth table of the logic gate of the device obtained through testing. The meaning of the “AND” logical operation state is the output is “1” only when all input signals are “1”; otherwise, it is “0”. The meaning of the “OR” logical operation state is as long as one input signal is “1”, the output will be “1”. Based on this principle, by introducing two types of input signals in thin-film transistors, two different logical operations, “AND” and “OR”, can be achieved. It is better than other optoelectronic synaptic devices shown in Table 1. The logic operation diagrams of “OR” and “AND” are shown in Figure 6c and d, respectively.

Among the information obtained by the human brain, the information transmitted by the visual system accounts for approximately 80%. The artificial synaptic device has two input signals: gate voltage and light pulse. The excitatory postsynaptic current of the device when V_GS_ was 0 V has been tested in the previous text. Therefore, the influence of the attention and visual systems on the memory level was simulated by using the two input signals. V_GS_ is 0 V indicates that the brain is in a state of inattention. V_GS_ is 16 V indicates that the brain is in a state of concentration. When there is no light pulse, it indicates that memory is not carried out through the visual system. When there is a light pulse, it indicates that memory is carried out through the visual system. The current value of the logical function test represents the memory level. While realizing the logical function, it also simulates the influence of different synaptic states on memory. It can be seen from the current values in Figure 6b that the memory levels in different states are different. The memory level is the highest when the attention is concentrated and the visual system is involved in the memory, and it has a more significant improvement compared to the first three states.

## 3. Conclusions

In this paper, a ZnAlSnO thin-film transistor type artificial neural synapse with a simple process and good electrical performance is successfully fabricated. Under the stimulation of light with a wavelength of 365 nm, the basic characteristics of the synapse, including EPSC, PPF, and synaptic plasticity, are well simulated. This device shows good synaptic plasticity. In addition, the channel current of the device at different gate voltages was tested by adjusting the gate voltage. When V_GS_ was 0 V, the average dark current was 3.17 μA, and when V_GS_ was 16 V, the average dark current was 4.48 μA, which was close to the current peak value triggered by the time pulse when V_GS_ was 0 V. When the photoelectric co-input was used, the peak value of the triggered current was significantly increased compared with the first three input states. Thus, through the photoelectric co-regulation, two different logical operations of “AND” and “OR” were achieved, and the influence of different synaptic states on memory was simulated. The AZTO thin-film transistor type artificial synapse prepared experimentally provides a new artificial synapse device solution for the development of high-performance memory and computing integrated architectures. More importantly, it offers a brand-new design idea for simplifying programmable neuromorphic logic circuits. This innovative method is expected to promote the development of neuromorphic computing hardware towards greater efficiency and flexibility.

## Figures and Tables

**Table 1:** Synaptic device performance comparison table.

Active Layer	Wavelength (nm)	Optical Density (mW/cm^2^)	Electrical Pulse Amplitude (V)	PPF Index (%)	Energy Consumption (pJ)	Logic Function	Ref.
AZTO	365	3.66	—	125.34	~10^6^	√	this work
IGZO	360–380	0.60	—	—	~10^5^	×	[14]
IGZO	405	0.182	—	176.9	—	×	[33]
IAZO	375	0.1	—	155.9	2.3	×	[25]
IGZO	UV light	3	—	70	—	×	[5]
IZO/IGZO	—	—	3.5	149.2	2.69 × 10^−4^	×	[9]
IGZO	—	—	1.4	150	975	×	[10]
IZO	—	—	0.5	156	~10^3^	×	[11]

## Data Availability

The original contributions presented in this study are included in the article. Further inquiries can be directed to the corresponding authors.
